# Factors Associated with Telemedicine Services Provision for Sexually Transmitted Disease Diagnosis and Treatment Among Dermatologists: Evidence from China

**DOI:** 10.1089/tmr.2022.0021

**Published:** 2022-09-06

**Authors:** Yingzhe Yu, Zhuo Chen, Jing Zhang, Ping Zhou, Lingyi Lu, Bingjiang Lin, Yang Li

**Affiliations:** ^1^ Department of Dermatology, Ningbo First Hospital, Ningbo, China.; ^2^ School of Economics, Faculty of Humanities and Social Sciences, University of Nottingham Ningbo China, Ningbo, China.; ^3^ Department of Health Policy and Management, University of Georgia, Athens, Georgia, USA.

**Keywords:** telemedicine, physician's perception of telemedicine, sexually transmitted diseases

## Abstract

**Background:**

Telemedicine has experienced rapid growth in China, with wide applications for chronic disease management.

**Objective:**

This study examined a unique survey dataset to identify the provision of telemedicine services by dermatologists, and to explore its association with physician characteristics, perception of diagnosis, and physicians' perceptions of the advantages and disadvantages of telemedicine.

**Materials and Methods:**

Responses to an anonymous voluntary questionnaire were collected from 238 dermatologists in Zhejiang Province, China, via a mixed mode of online and in-person data collection. Data were analyzed using Stata 16.0. Empirical analyses utilized descriptive statistics and multivariable logistical regression.

**Results:**

Among a total of 238 physicians, 34.9% provided telemedicine services. Results from the multivariable logistic regression indicated that, if physicians can use their spare time to help patients, seniority and their perception of the benefit of telemedicine are the two most important factors determining their likelihood of providing telemedicine services among the studied sample.

**Conclusion:**

Telemedicine holds great promise, but its practices need to be more efficient to save time and reduce the risk of misdiagnosis so that more physicians may participate.

## Introduction

Telemedicine is the provision of medical care remotely through audiovisual technologies. With telemedicine, clinicians can examine patients, provide diagnoses, and recommend treatment options from a long distance.^1^ With the popularity of the Internet and smartphones, China's telemedicine has had significant development. Since the outbreak and spread of the COVID-19 epidemic, telemedicine utilization has seen a period of accelerated growth.

The rising medical costs has been an immense challenge for the Chinese government. In 2009, the increase in the government's financial support in health was one of the main initiatives in the new round of medical and health system reform launched by the Chinese government. However, from 2009 to 2017, China's total health expenditure increased from 17.5 trillion yuan to 52.6 trillion yuan, and an average annual growth rate of per capita spending on health care at 14.75%.^2^ A prior study had shown that telemedicine, for example, Internet health care, can play an important role in reducing medical costs, especially for tertiary hospitals.^2^

Meanwhile, the growing disparities in the distribution of high-quality health care resources continue to be one of the most pressing social issues. Of relevance to the distribution are non-medical factors, including the city's administrative level and power. Studies have shown that Tier 3-A hospitals were concentrated in the major municipalities, provincial capitals, and coastal cities.^3,4^ Medical resources have a severely unequal distribution across different social groups in China, which led to barriers to accessing quality medical resources.^5^ Telemedicine could reduce barriers in accessing care because of travel distance or inability to travel due to disability, in addition to the uneven distribution of medical resources.^6^

Internet hospitals, the online platform to provide telemedicine services to patients, began to emerge in 2014 in China.^7^ By 2018, according to the requirements of the General Office of the State Council, “Opinions on Promoting the Development of ‘Internet + Medical Health,’” China's National Health Commission issued a series of supporting documents on July 17, 2018, for example, “Internet Diagnosis and Treatment Management Measures (Pilot),” “Internet Hospital Management Measures (Pilot),” and “Telemedicine Service Management Specifications (Pilot).”^8^

The government promulgated specific regulations on Internet hospital management, including enrolling physicians, electronic prescriptions, and other aspects relevant to telemedicine. It marked the official beginning of the era of standardized development of Internet hospitals in China. Meanwhile, the government had devoted efforts to improving the medical insurance reimbursement policy for Internet medical treatment. At present, online settlement of medical insurance is possible in some Internet hospitals in Jiangsu, Shanghai, and other places.^9^

With the aforementioned policies, there were 711 Internet hospitals in Mainland China by July 16, 2020, including brick-and-mortar hospitals offering telemedicine. The number of Internet hospitals increased to more than 1600 in early 2021.^9^ Accordingly, more than 70% of adult patients had sought medical information online.^10,11^ Telemedicine has been used in chronic disease management. Its primary roles have been in providing health education (to improve self-management), enabling clinical surveillance (e.g., telemonitoring), facilitating communication with health professionals (e.g., telephone support and follow-up), and improving electronic records.^12^

Available statistics showed that, among the online medical consultations, the dermatology department was one of the most utilized departments.^11^ In China, the dermatology department played an instrumental role in the prevention and control of sexually transmitted diseases (STDs), such as syphilis. Patients are increasingly used to gathering and gleaning information through the Internet.^13^ Due to the stigmatization associated with STD, STD patients may have a higher demand for telemedicine than other patients because telemedicine visits offer better privacy protection than onsite visits in China.

An increasing body of studies has explored patients' demand for telemedicine, and telemedicine interventions have a broad, multisectoral field of application.^14^ It could serve different populations. For instance, with the help of telemedicine, pregnant women were less likely to experience gestational weight gain and more likely to quit smoking.^15^ Moreover, the users who mainly used the Internet for health purposes were women of younger age and those with higher educational levels.^16^

Digital health interventions have benefited children and adolescents with mental health problems.^17^ Telemedicine was also increasingly becoming a reality in medical care for older adults. For cardiovascular disease or diabetes, older adult patients who use telemedicine devices tend to have better results for “behavioral” endpoints such as adherence to medication or diet and self-efficacy.^18^

However, a few studies have examined physicians' perspectives on using telemedicine among dermatologists. According to Ly et al, in Senegal, 72.1% of the physicians working in public hospitals and 82.1% of the physicians working in district health centers were likely to use telemedicine in their professional activities.^19^ Little attention has been devoted to physicians' provision of telemedicine services in China.^20–22^ To the best of our knowledge, this study is the first to explore Chinese physicians' motivation and behaviors in providing telemedicine services in instances of STDs, using a unique survey dataset.

## Materials and Methods

### Study setting

As STDs are mainly diagnosed and treated in dermatology departments in China, this survey was conducted among a convenience sample of 238 dermatologists from 96 hospitals in Zhejiang Province, China. The 96 hospitals are located in 11 cities in Zhejiang Province. Responses from 238 participants were collected from January 12, 2022 to February 12, 2022. The anonymous questionnaires (see Supplementary data) were sent to the dermatologists both online and in person.

The Ethics Committee of Ningbo First Hospital, Zhejiang Province, China, approved the ethical assessment of the study (No. 2021-R182), and verbal informed consent was obtained from all study subjects.

### Questionnaire development

The questionnaire developed by the research team consisted of five parts: general information (rating of hospital, years of working experience, and professional seniority), condition of continuing health education, diagnosis and treatment protocol, internet medical care engagement, and attitudes toward online medical treatment. The survey aimed at fully understanding dermatologists' perception of the advantages and disadvantages of telemedicine.

The diagnosis and treatment protocol focus on partner notification, attention for further contact, providing health education to patients, and informing patients of follow-up plans and the infectious disease report card.

### Statistical analysis

Descriptive statistics, including means, standard deviations, and percentages, are used to describe the occurrence of online medical consulting by physicians, physician information, and the advantages and disadvantages of providing telemedicine among the sample.

Multivariable logistic regression models were used to explore the key variables that affect physicians' provision of telemedicine services. Odds ratios (OR) and 95% confidence intervals (95% CI) were estimated for the association between independent variables (physician perceptions) and the dependent variable (telemedicine provision patterns). All analyses were conducted using Stata 16.0.

## Results

### Summary statistics

Table 1 illustrates the summary statistics from the physician questionnaire. In our sample, 60.1% of physicians are from tertiary hospitals, and physicians in the secondary and primary hospitals account for 24.8% and 6.3% of the total observations, respectively. As a secondary specialty, only a few primary hospitals have dermatology departments. Therefore, dermatologists are more likely to be in secondary and tertiary hospitals.^23^

**Table 1.** Summary Statistics

	Proportion/mean	Standard deviation
Hospital level		
Primary hospital	0.063	
Secondary hospital	0.248	
Tertiary hospital	0.601	
Specialized hospital	0.088	
Physician seniority		
Resident	0.223	
Fellow	0.361	
Associate chief physician	0.282	
Chief physician	0.134	
Online medical consulting	0.349	
Continuing health education	0.882	
Actively screened for patients with potential STDs	0.971	
Work years	14.52	10.39
Observations	238	

STDs, sexually transmitted diseases.

Our sample is a good representation of dermatologists across different levels of hospitals in Zhejiang. Regarding the physician career stage, 22.3% of physicians in our survey are residents, and 36.1% of physicians are fellows. The proportion of associate chief physicians and chief physicians is 28.2% and 13.4%, respectively. The next panel shows the information regarding work experience and online medical consulting. The physicians in our sample, on average, have 14.5 years of working experience. Among them, 83 physicians (34.9%) have provided online medical consulting. As for their professional knowledge of STDs, 88.2% have participated in continuing health education, and 97.1% have actively screened for patients with potential STDs.

Table 2 reports reasons for reluctance to provide online medical treatment by hospital levels. Overall, 65% ( = 155/238 × 100%) physicians did not have any experience in providing telemedicine services. Among them, 88 physicians are from tertiary hospitals in Zhejiang. Physicians in primary hospitals did not provide online medical treatment mostly because they think telemedicine is more likely to have medical misdiagnosis (60%) and is less efficient (60%). Except for primary hospitals, most physicians in higher tiers of hospitals have not provided telemedicine services because they are occupied with their job and do not have enough time. The proportion is 59% in secondary hospitals, 73.9% in tertiary hospitals, and 66.7% in specialty hospitals, respectively.

**Table 2.** Reasons for Reluctance to Use Telemedicine by Hospital Level

	Primary hospital	Secondary hospital	Tertiary hospital	Specialized hospital
Have no time	0.500	0.590	0.739	0.667
Lack of access	0.200	0.359	0.205	0.389
Easy to misdiagnose	0.600	0.538	0.568	0.556
Low efficiency	0.600	0.128	0.136	0.222
Observations	10	39	88	18

Table 3 illustrates why physicians have little incentive to provide online medical treatment by career stage. The primary reason is that they did not have enough time for telemedicine, especially for senior physicians.

**Table 3.** Reasons for Reluctance to Use Telemedicine by Physician Seniority

	Resident	Fellow	Associate chief physician	Chief physician	All
Have no time	0.463	0.691	0.767	0.938	0.677
Lack of access	0.268	0.218	0.302	0.313	0.265
Prone to misdiagnose	0.439	0.600	0.581	0.688	0.561
Low efficiency	0.220	0.200	0.116	0.125	0.174
Observations	41	55	43	16	155

Table 4 shows the advantages of online medical treatment by physician seniority. Physicians across different positions all share quite similar opinions on the advantages of telemedicine. Overall, about 90% of physicians consider online medical treatment more convenient for patients. Approximately 75% of physicians report that the advantage of telemedicine is to provide health education to patients more conveniently. About 71% of physicians consider telemedicine beneficial, because they can use spare time to help patients.

**Table 4.** Advantages of Telemedicine by Physician Career Stage

	Resident	Fellow	Associate chief physician	Chief physician	All
More convenient for patients	0.943	0.907	0.851	0.969	0.908
Regular follow-up	0.717	0.674	0.507	0.688	0.639
Provide health education more conveniently	0.830	0.791	0.657	0.719	0.752
Use spare time to solve problems for patients	0.811	0.686	0.657	0.719	0.710
Protect patient privacy	0.830	0.581	0.612	0.844	0.681
Observations	53	86	67	32	238

Table 5 illustrates the disadvantages of telemedicine. Similar preferences are shared across different levels of physicians. On average, about 96% of physicians consider as a disadvantage of online medical treatment the inability to perform a physical examination (PE) intuitively and correctly. Nearly 80% of them also think that telemedicine leads to lower consultation efficiency.

**Table 5.** Disadvantages of Telemedicine by Physician Seniority

	Resident	Fellow	Associate chief physician	Chief physician	Total
Low efficiency of consultation	0.868	0.837	0.716	0.750	0.798
Unable to perform PE intuitively	0.981	0.942	0.955	1	0.962
Take up free time	0.528	0.523	0.552	0.625	0.546
Observations	53	86	67	32	238

PE, physical examination.

### Empirical analysis

We adopted the multivariable logistic regression models to explore the determinants that affect physicians' telemedicine services provision. The empirical analysis aims at identifying the factors that determine the provision of telemedicine services with a logistic regression:

(1) $$Y_{ij}=\beta_{1}+\beta_{2}\gamma_{j}+\beta_{3}X_{1ij}+\beta_{4}X_{2ij}+\beta_{5}X_{3ij}+\delta_{ij}$$

In regression (1), iindexes physician, *j* indexes hospital level.$Y_{ij}$ is a binary variable, indicating whether physician *i* had provided telemedicine services to patients. $\gamma_{j}s$ are hospital fixed-effects. The vector $X_{1ij}$, $X_{2ij}$and $X_{3ij}$ represent a set of key independent variables, which varies across different specifications. For instance, $X_{1ij}$ includes physicians' information such as position seniority and working years; $X_{2ij}$ indicates physicians' perceptions of the importance of STDs diagnosis.

$X_{3ij}$ contains variables that reflect physicians' attitudes toward telemedicine services. The coefficient of interest $\beta_{3}$ estimates the association between independent variables and dependent variables (whether provided telemedicine services). $\delta_{ij}$ is the residual term.

The results of multivariable logistic regression analyses are presented in Table 6, with odds ratios and 95% CI estimated and reported. Column (1) displays the baseline model, with physician information as the key independent variables. Columns (2) and (3) include physicians' perceptions of STDs diagnosis and their telemedicine preference, respectively. Column (4) includes all key independent variables. We include hospital-level fixed effects in all specifications to control for time-invariant systematic differences in physician characteristics across hospital types.

**Table 6.** Estimated Results of Determinants of Telemedicine Services Provision from Multivariable Logistic Regression Models

	(1)	(2)	(3)	(4)
Physicians' information				
Physician seniority (resident as reference group)				
Fellow	2.539^**^ (1.049–6.150) 0.0389	2.373^*^ (0.952–5.912) 0.0636	3.870^***^ (1.490–10.05) 0.00544	3.488^**^ (1.306–9.319) 0.0127
Associate chief	2.793^*^ (0.939–8.307) 0.0648	3.367^**^ (1.100–10.31) 0.0335	4.423^**^ (1.384–14.14) 0.0121	4.941^***^ (1.507–16.20) 0.00837
Chief physician	6.301^**^ (1.491–26.62) 0.0123	6.693^**^ (1.533–29.22) 0.0115	10.35^***^ (2.263–47.31) 0.00259	10.57^***^ (2.239–49.90) 0.00290
Work year	0.980 (0.939–1.022) 0.345	0.972 (0.930–1.015) 0.201	0.968 (0.925–1.012) 0.155	0.962 (0.918–1.008) 0.102
Perception of STDs diagnosis				
Partner notification		1.890 (0.837–4.271) 0.126		1.743 (0.745–4.080) 0.200
Further contact		1.686 (0.876–3.245) 0.118		1.429 (0.715–2.855) 0.312
Provide health education		1.610 (0.367–7.070) 0.528		1.470 (0.328–6.576) 0.615
Inform follow-up plan		2.813 (0.638–12.40) 0.172		3.711^*^ (0.785–17.54) 0.0980
Disease report card		0.721 (0.0937–5.541) 0.753		0.339 (0.0412–2.785) 0.314
Telemedicine preference				
More convenient			1.154 (0.379–3.510) 0.801	0.941 (0.297–2.982) 0.918
Regular follow-up			0.745 (0.346–1.602) 0.451	0.729 (0.330–1.610) 0.434
Provide health education			1.254 (0.518–3.037) 0.615	1.245 (0.490–3.160) 0.645
Use spare time			4.046^***^ (1.808–9.052) 0.000670	3.964^***^ (1.748–8.989) 0.000979
Privacy			1.351 (0.635–2.875) 0.435	1.309 (0.603–2.841) 0.496
Constant	0.292^*^ (0.077–1.104) 0.0695	0.0652^**^ (0.007–0.629) 0.0183	0.0495^***^ (0.007–0.328) 0.00183	0.0307^***^ (0.002–0.400) 0.00784
Observations	238	238	238	238
Hospital FE	Yes	Yes	Yes	Yes

Numbers in parentheses are 95% CI. *p*-Values are shown below 95% CI, with significance level as follows: ^***^p < 0.01, ^**^p < 0.05, and ^*^p < 0.1.

CI, confidence intervals; FE, fixed effect.

For physician information, the odds ratios of physician seniority increase along with the seniority and always larger than 1. For instance, in column (1), the odds ratios range from 2.539 (95% CI: 1.049–6.150) for Fellows to 6.301 (95% CI: 1.491–26.62) for Chief Physicians, and the coefficients of physician seniority are all statistically significant (*p*-value* < 0.1) in four specifications. This result indicates that a physician's career stage has a significantly positive effect on the provision of online medical treatment, that is, physicians with a higher position are more likely to provide telemedicine services. This may be explained by the fact that senior physicians, such as Chief Physicians, are more likely to be trusted by patients and thus more welcome among patients who seek diagnoses and advice online.

Regarding the perception of STD diagnosis illustrated in Column (2), all independent variables are statistically insignificant. Therefore, physicians' provision of telemedicine services is not affected by their perception of STDs diagnosis.

Column (3) displays the results of physician attitudes toward telemedicine services. *Use of spare time* (OR: 4.046, 95% CI: 1.808–9.052), among all the advantages of telemedicine, is the most important reason affecting physicians' telemedicine provision decisions. Physicians who consider telemedicine beneficial because they can use spare time to treat patients have a higher probability of using telemedicine.

Column (4) reports the results with all key independent variables. The findings are similar to the earlier mentioned analysis and results, with one exception—after controlling for physician telemedicine preferences, *Inform follow-up plan* (OR: 3.711, 95% CI: 0.785–17.54) has a significant positive impact on the probability of providing online medical treatment. Therefore, physicians who informed patients of follow-up plans after diagnosis are more likely to provide telemedicine services than physicians who did not inform follow-up plans after we included physicians' telemedicine preferences.

To sum up, the provision of telemedicine services is affected by physician position and physicians' attitudes toward telemedicine services.

## Discussion

### Summary of findings

To examine the factors related to physicians' use of telemedicine in China, our study used a well-designed physician survey dataset with a rigorous econometric framework. To the best of our knowledge, this study is the first to use the framework in examining the individual and contextual factors that determine the provision of telemedicine services in China. We explored the association between physician information, perception of STD diagnosis, and attitudes toward telemedicine services with the occurrence of physicians' provision of telemedicine services.

Overall, there were several factors associated with the patterns of telemedicine services provision. In particular, the results from the multivariable logistic regression model highlight the relevance of physician seniority and physicians' perceptions of the benefit of telemedicine, specifically that they can make use of their spare time to help patients, are the two most important factors in understanding their likelihood in using telemedicine among the studied sample.

### Advantages of telemedicine

As discussed in Table 4, physicians across different positions all share similar opinions on the advantages of telemedicine. Overall, most physicians consider telemedicine more convenient for patients, it easier to provide health education, and they can use their spare time to provide patients diagnoses and advice. These findings are consistent with prior research. There are three prominent domains of telemedicine: (1) using eHealth technologies to monitor, track, and inform health; (2) using digital technologies to enable health communication between health professionals and patients; and (3) data enabling health-collecting, managing, and using health data.^24^

Due to its characteristics, telemedicine has been highly focused on during the time of coronavirus. Not only can telemedicine provide medical care to patients while reducing the risk of COVID-19 transmission among patients, family members, and clinicians, but it can also allow physicians and patients to communicate 24/7, using smartphones or webcam-enabled computers.^25^ Local epidemiologic information can be used to standardize screening and practice patterns across providers.^26^

### Disadvantages of telemedicine

For the disadvantages of telemedicine, most physicians in our sample question the efficiency of telemedicine because physicians think they may not be able to perform a PE intuitively and with accuracy. They also consider telemedicine prone to have lower efficiency of consultation.

Our assessment of the disadvantages of telemedicine broadly affirms those of prior studies, including those undertaken primarily in high-income countries. The acceptance of eHealth interventions was rather low in inpatient routine care, and there is a considerable number of people questioning the effectiveness and safety of digital health.^27^ Further, we would risk wasting scarce health care resources on ineffective programs if there is no clear evidence regarding when and where telemedicine is most effective. In addition, the legal and regulatory infrastructure for telemedicine has yet to catch up with the development of technology, which evolves on a near-daily basis.^1,28^

## Conclusion

Telemedicine has significantly improved access to medical care. With the rapid development of telemedicine, it is important to have enough doctors to participate in telemedicine provision. Meanwhile, the perception and the attitude of physicians should be examined. Our findings suggest that improving telemedicine's efficiency should be a top priority. Because of the compact work schedule of medical professions, physicians have limited spare time for telemedicine provision:

It is necessary to have a simple and efficient route to set up telemedicine, for example, establishing a standardized consultation mode to reduce ineffective communication. On the other hand, how to reduce misdiagnosis is critical. Dermatologists in our survey, who usually provide diagnosis using the picture of clinical manifestations during telemedicine, are more concerned about misdiagnosis. However, this study has two important limitations.

First, the convenience sampling used in the study suffers from potential sampling bias. Second, the number of survey respondents was limited due to the restrictions imposed by the COVID-19 prevention and control measures during the study period. We hope that there will be more future research in the area and, with the development of technology, including artificial intelligence, telemedicine will be able to substantially improve patient health and satisfaction as mentioned earlier.

## Authorship Contribution Statements

Y.Y.: Conceptualization, investigation, writing-original draft preparation, writing—review and editing, and funding acquisition. Z.C.: Methodology, writing—review and editing, and supervision. J.Z.: Investigation, data curation. P.Z.: conceptualization, supervision. L.L.: investigation, data curation. B.L.: investigation, supervision. Y.L.: conceptualization, methodology, formal analysis, writing-original draft preparation, and writing-review and editing. All authors have read and agreed to the published version of the article.

## Supplementary Material

Supplemental data

## Abbreviations Used

CI: confidence intervals
OR: odds ratio
PE: physical examination
STDs: sexually transmitted diseases

## Notes

^*^
In our paper, the null hypothesis states that there is no relationship between dependent variables and the independent variable tested. We adopt the Z-test statistics to test this hypothesis. The P-value is used as an alternative to the rejection point to provide the least significance at which the null hypothesis would be rejected. If the P-value is small, then there is stronger evidence in favor of the alternative hypothesis.
