# Supplementary material for: Factors Associated with Telemedicine Services Provision for Sexually Transmitted Disease Diagnosis and Treatment Among Dermatologists: Evidence from China
Source: Telemed Rep. 2022 Sep 6;3(1):166–73. doi: 10.1089/tmr.2022.0021 (PMC9531886; doi:10.1089/tmr.2022.0021)
Supplement: Supplemental data [file Supp_Data.docx]

**Questionnaire**

Hello, our team is currently carrying out a survey of the current situation of syphilis diagnosis and treatment and perception of telemedicine for dermatologists. We appreciate your time and hope you can complete the questionnaire to provide important information for research. Thank you!

PART1 General Information

1. **Institution [fill in the blank]** _________________________

**Level of hospital**

1. Tertiary Hospital 2. Secondary Hospital 3. Primary Hospital 4. Others

**Grade of hospital：**

1. Grade A 2. Grade B 3. No Grade

**2. Position title [****single answer]**

1. chief physician

2. associate chief physician

3. fellow

4. resident

5. assistant physician

**3. Working years [fill in the blank]** _____________years

PART2 Condition of continuing health education

**4. Have you participated in online and offline continuing education activities related to sexually transmitted diseases（STDs）within 3 years? [single** **answer]**

0. No 1.Yes

PART3 Diagnosis and treatment protocol

**5. Did you actively screen for patients with potential STDs，who have suspicious symptoms or are high-risk groups in your clinical work? [single answer]**

0. No 1.Yes

**6.Did you inform their sexual partners to take the STDs test?**

0. No 1.Yes

**7.Did you pay more attention to further contact?**

0. No 1.Yes

**8.Did you offer health education for patients and provision of relevant health promotion?**

0. No 1.Yes

**9.Did you inform patients of the follow-up plan?**

0. No 1.Yes

**10.Did you submit infectious disease report card?**

0. No 1.Yes

**11. Do you have a stigma impression or discrimination against syphilitic patients [single answer]**

0. No 1.Yes

PART4 Internet medical care engagement

**12. Have you ever participated in online medical consulting? (including hospital Internet platform, the public health service platform of government, HaoDaifu online, DXY, etc.) [ single answer]**

0. No 1.Yes

**If** **the answer to question 8 is "yes"，please answer questions 9 and 10; If the answer to question 8 is "no", please answer questions 11 and 12.**

**13. The frequency of** **telemedicine work is about [single answer]**

1. more than 1 person per day

2. less than 1 person per day, more than 1 person per week

3. less than 1 person per week, more than 1 person per month

4. less than 1 person per month

5. Others_________________

**14. How about the follow-up of online patients? [single answer]**

1. similar to inperson patients

2. significantly less than inperson patients

3. significantly more than inperson patients

4. Others_________________

PART5 Attitudes towards online medical treatment

**15. Factors affecting your provision of telemedicine** **(multiple answers):**

1. Have no time, too busy with routine work

2. Lack of access to telemedicine

3. Easy to misdiagnose during telemedicine

4. Low efficiency of telemedicine

5. Others_________________

**16. Are you willing to participate in telemedicine if you have the opportunity?** **[single answer]**

0. No 1.Yes

**17. Do you think it is necessary to offer online care, for example, send reminders via app or text message? [single answer]**

0. No 1.Yes

**18. What do you think are the advantages of telemedicine? (multiple answers)**

1. More convenient for patients

2. Provide regular follow-up

3. Provide patients with relevant health education more conveniently and reliably

4. Use spare time to solve problems for patients

5. Protect patient privacy

6. Others_________________

**19. What do you think are the disadvantages of telemedicine? (multiple answers)**

1. Low consultation efficiency because of unclear expression or concealment of the actual situation from patients

2. Unable to conduct physical examination directly which affects diagnosis

3. Occupy personal spare time

4. Others_________________

PART6 Knowledge of sexually transmitted diseases

**The following are 10 questions related to syphilis diagnosis and treatment, please determine whether the description is right or wrong based on your understanding.**

**20. Early syphilis refers to being infected with Treponema pallidum within 2 years, including primary, secondary and early latent syphilis.**

1. Right 0. Wrong

**21. The clinical manifestations of secondary syphilis include generalized superficial enlarged lymph nodes, ocular syphilis, polymorphic mucocutaneous lesions and gumma.**

1. Right 0. Wrong

**22. If the infection was less than 4 weeks, Treponema pallidum serologic tests could be negative.**

1. Right 0. Wrong

**23. Early syphilis is highly contagious. Therefore, it is recommended to consider preventive anti-syphilis treatment in patients with sexual contact within 3 months, regardless of the results of serologic tests.**

1. Right 0. Wrong

**24. If the non-Treponema pallidum serologic test changes from negative to positive or the titer increases by more than 4 times compared with the previous time, it is a clinical relapse.**

1. Right 0. Wrong

**25. 3 to 6 months after the end of early syphilis treatment, the treatment is considered to be effective if the titer of non-Treponema pallidum serological test decreases by 2 times or more than before.**

1. Right 0. Wrong

**26. Commonly used drugs for syphilis treatment are benzylpenicillin, procaine penicillin, ceftriaxone, doxycycline, macrolides.**

1. Right 0. Wrong

**27. Jarisch-Herxheimer reaction can occur after treatment for syphilis, which often occurs several hours after the primary doses of** **antisyphilitic treatment and fades within 24 h.**

1. Right 0. Wrong

**28.** **Syphilitic patients have undergone standard antisyphilitic treatment and adequate follow-up, and the** **non-Treponema pallidum serologic test has been maintained at a certain titer (usually 1:8 or below) for more than 3 months, excluding neurosyphilis, cardiovascular syphilis, etc., that is,** **syphilis sero-resistance.**

1. Right 0. Wrong

**29. No further treatment is required for** **syphilitic patients with sero-resistance if the titer of non-syphilitic spirochete serological test was increased by four times.**

1. Right 0. Wrong
